# Self-Nanoemulsifying Drug Delivery System (SNEDDS) of Apremilast: In Vitro Evaluation and Pharmacokinetics Studies

**DOI:** 10.3390/molecules27103085

**Published:** 2022-05-11

**Authors:** Ahad S. Abushal, Fadilah S. Aleanizy, Fulwah Y. Alqahtani, Faiyaz Shakeel, Muzaffar Iqbal, Nazrul Haq, Ibrahim A. Alsarra

**Affiliations:** 1 Department of Pharmaceutics, College of Pharmacy, King Saud University, Riyadh 11451, Saudi Arabia; ahad.abushal@gmail.com (A.S.A.); faleanizy@ksu.edu.sa (F.S.A.); fyalqahtani@ksu.edu.sa (F.Y.A.); fsahmad@ksu.edu.sa (F.S.); nhaq@ksu.edu.sa (N.H.); 2 Department of Pharmaceutical Chemistry, College of Pharmacy, King Saud University, Riyadh 11451, Saudi Arabia; muziqbal@gmail.com; 3 Central Laboratory, College of Pharmacy, King Saud University, Riyadh 11451, Saudi Arabia

**Keywords:** apremilast, psoriatic arthritis, pharmacokinetics studies, SNEDDS, solubility, dissolution, oral bioavailability

## Abstract

Psoriatic arthritis is an autoimmune disease of the joints that can lead to persistent inflammation, irreversible joint damage and disability. The current treatments are of limited efficacy and inconvenient. Apremilast (APR) immediate release tablets Otezla^®^ have 20–33% bioavailability compared to the APR absolute bioavailability of 73%. As a result, self-nanoemulsifying drug delivery systems (SNEDDS) of APR were formulated to enhance APR’s solubility, dissolution, and oral bioavailability. The drug assay was carried out using a developed and validated HPLC method. Various thermodynamic tests were carried out on APR-SNEDDS. Stable SNEDDS were characterized then subjected to in vitro drug release studies via dialysis membrane. The optimum formulation was F9, which showed the maximum in vitro drug release (94.9%) over 24 h, and this was further investigated in in vivo studies. F9 was composed of 15% oil, 60% S_mix_, and 25% water and had the lowest droplet size (17.505 ± 0.247 nm), low PDI (0.147 ± 0.014), low ZP (−13.35 mV), highest %T (99.15 ± 0.131) and optimum increases in the relative bioavailability (703.66%) compared to APR suspension (100%) over 24 h. These findings showed that APR-SNEDDS is a possible alternative delivery system for APR. Further studies are warranted to evaluate the major factors that influence the encapsulation efficiency and stability of APR-containing SNEDDS.

## 1. Introduction

Psoriasis is a well-known chronic inflammatory autoimmune disease of the skin that occurs in 2–4% of the world’s population. Both psoriasis and psoriatic arthritis are remitting and relapsing diseases [1,2]. The co-existence of environmental factors or stress factors can trigger the onset of psoriatic arthritis. The treatment of psoriatic arthritis is based on the initial assessment of the disease severity, which is determined by the degree of inflammation, pain, the number of joints involved, and the degree of disability. The treatment can be achieved using single or multiple drug therapies depending on the disease stage and considering the patients’ preference (route of administration, frequency and side effects tolerability) [3]. The currently used treatment regimens involve non-steroidal anti- inflammatory drugs (NSAIDs), intra-articular corticosteroid injections, disease modifying antirheumatic drugs (DMARDs), and biologics [4,5].

NSAIDs are mostly used for mild psoriatic arthritis alone or combined with other agents like intra-articular corticosteroid injections. This combination is used for symptomatic relief only; it creates synergetic anti-inflammatory action since they act on different inflammatory pathways [6,7]. They do not alter the disease progression course and their side effects are not well tolerated by most patients, especially their gastric adverse events [5,6]. DMARDs are more effective in treating psoriatic arthritis than NSAIDs or intra-articular corticosteroid injections since they can not only improve the inflammatory symptoms, but also reduce the progressiveness of the disease, which improves patient’s health-related quality of life [4]. Biologics are the most expensive treatment option; despite that, they are still used because many studies proved that they are the most effective in treating psoriatic arthritis and are superior in overcoming the inflammation symptoms and pain, minimizing the progression of the diseased joints, and enhancing the quality of life of patients when compared to the other remedies. Unfortunately, they have a major drawback as they lose their efficacy during the treatment course as the body produces antibodies against them [5].

Apremilast (APR) is the first orally administered drug approved for the treatment of active psoriatic arthritis in adults. It was also assigned for the treatment of dermatologic psoriasis and many other diseases such as rheumatoid arthritis, atopic dermatitis and Beçhet’s syndrome [5]. It belongs to a group of drugs known as cyclic nucleotide phosphodiesterase type-4 (PDE-4) inhibitors. In comparison to the common treatment regimens, treatment with APR was tolerated by patients and associated with better overall disease-related improvements. APR is a class IV drug, which means it has poor water solubility and permeability, hence it has poor rate of dissolution and consequently poor oral bioavailability [8,9]. Very limited formulation approaches were found in the literature to enhance APR solubility and oral bioavailability [10,11]. The marketed film coated immediate release tablets of APR Otezla^®^ have 20–33% bioavailability compared to APR absolute bioavailability of 73% [5]. On the other hand, many studies represented SNEDDS as promising delivery systems for pharmaceutical drugs due to their tremendous advantages in enhancing solubility, spontaneously occurring emulsification, and thermodynamic and kinetic stabilities [12,13,14,15,16,17,18]. The aim of the present study was to create and optimize SNEDDS of APR to increase its solubility and dissolution rate, which sequentially will upgrade the extent of the oral bioavailability and therapeutic efficacy of the drug.

## 2. Results

### 2.1. Solubility Studies

The results of APR equilibrium solubility are illustrated in the table below (Table 1). The results of APR equilibrium solubility in water, oils, surfactants, and cosurfactants were greatly variable. The maximum equilibrium solubility of APR was observed in Transcutol-HP with a value of 55.01 ± 3.19 mg/mL followed by Tween-80 with a value of 48.54 ± 3.76 mg/mL; thus, these two components were further used as a surfactant and cosurfactant in APR-SNEDDS. The maximum oil solubility of APR was observed in Lauraglycol-FCC (36.54 ± 2.78 mg/mL) compared to the other oils; Lauroglycol-90 (28.21 ± 1.45 mg/mL), Capryol-PGMC (21.41 ± 1.32 mg/mL), Capryol-90 (18.15 ± 1.03 mg/mL), and Triacetin (11.42 ± 0.95 mg/mL); thus, Lauraglycol-FCC was selected as the oil phase in APR-SNEDDS. The least equilibrium solubilites were observed with water (0.01 ± 0.00 mg/mL), ethanol (0.66 ± 0.01 mg/mL) and IPA (2.07 ± 0.10 mg/mL) apparently due to poor APR hydrophilicity. However, water was preferred to be used as the aqueous phase in APR-SNEDDS due to its inert nature, high miscibility with the formulation component, high formulation compatibility and its frequent use in the literature [19,20]. 

### 2.2. Pseudo-Ternary Phase Diagrams for APR SNEDDS

**Figure 1:**
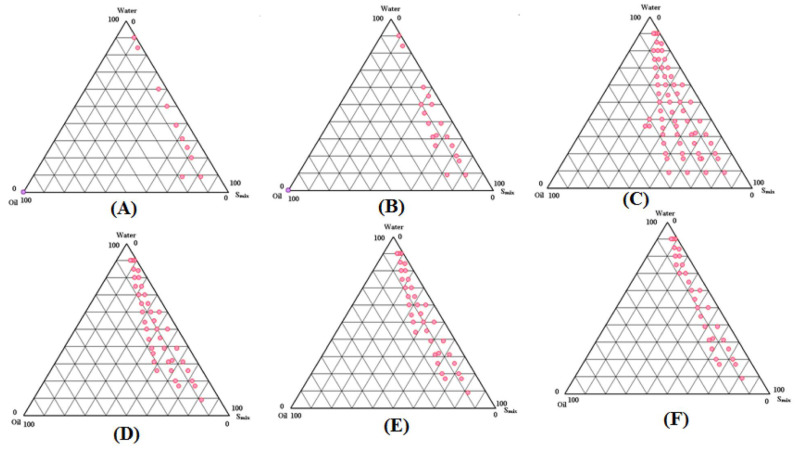
Pseudo-ternary phase diagrams showing SNEDDS zones for oil phase (Lauroglycol-90), aqueous phase (water), surfactant (Tween-80) and cosurfactant (Transcutol-HP) at S_mix_ ratios of (**A**) 1:0, (**B**) 1:2, (**C**) 1:1, (**D**) 2:1, (**E**) 3:1, and (**F**) 4:1.

A total of six phase diagrams were developed (Figure 1A–F); each contained different ratios of aqueous phase, oil phase and S_mix_. Depending on the S_mix_ ratios mainly, the first phase diagram (A) with (1:0) S_mix_ ratio showed the least emulsification areas. Next to it, was phase diagram (B) with (1:2) S_mix_ ratio, which showed very small emulsifications areas too. For phase diagrams (C) and (D) with (1:1) and (2:1) S_mix_ ratios respectively, the maximum emulsification areas were observed, but phase diagram (C) was superior to phase diagram (D) with slightly bigger emulsification areas. The last two phase diagrams (E) with (3:1) S_mix_ ratio and (F) with (4:1) S_mix_ ratio, showed moderate emulsification areas when compared to the least emulsification areas (A,B) and maximum emulsification areas (C,D) observed. From the above findings, phase diagram (C) with (1:1) S_mix_ ratio and the largest emulsification areas was chosen for APR-SNEDDS formulation development.

### 2.3. Formulation Development

After choosing 1:1 S_mix_ ratio of Tween 80 and Transcutol-HP (Phase diagram 1C), which gave the maximum nano-emulsification areas, nine APR-SNEDDS, namely (F1–F9), were developed. Each SNEDDS contained 5 mg of the drug in a total of 1 mL formulation. The formulations were prepared considering almost an entire range of SNEDDS zones in phase diagram with various Lauraglycol-FCC (oil phase) concentrations (10, 15, 20, 25% *v*/*v*), S_mix_ concentrations (30, 40, 45, 50, 55, 60% *v*/*v*) and de-ionized water (aqueous phase) concentrations (25, 30, 35, 40, 50, 55% *v*/*v*). The drug was dissolved completely in Lauraglycol-FCC before the addition of Tween 80: Transcutol-HP followed by titration with de-ionized water. The composition of APR SNEDDS is included in Table 2.

### 2.4. Thermodynamic Stability Testing

The formulations F3, F4, F7, F8 and F9 withstood the testing and showed no lack or loss of stability in terms of phase separation (flocculation, coalescence, phase inversion) or drug precipitation. The rest of the formulations F1, F2, and F6 were metastable and F5 was unstable (Table 3).

### 2.5. Self-Nanoemulsification Efficiency Test

The results for the self-nanoemulsification test are shown in Table 3. All APR-SNEDDS (F1–F9) were subjected to self-nanoemulsification efficiency test to assess their ability to maintain their stability upon dilution with aqueous phase at different pH values (neutral, acidic and basic). The efficiency of each formulation was evaluated and graded via visual inspection and the use of a system for grading. All the formulations (F1–F9) were graded as grade (A) as they rapidly formed clear NEs within 1 min and maintained their physical and thermodynamic stabilities. 

### 2.6. Physicochemical Characterization

**Figure 2:**
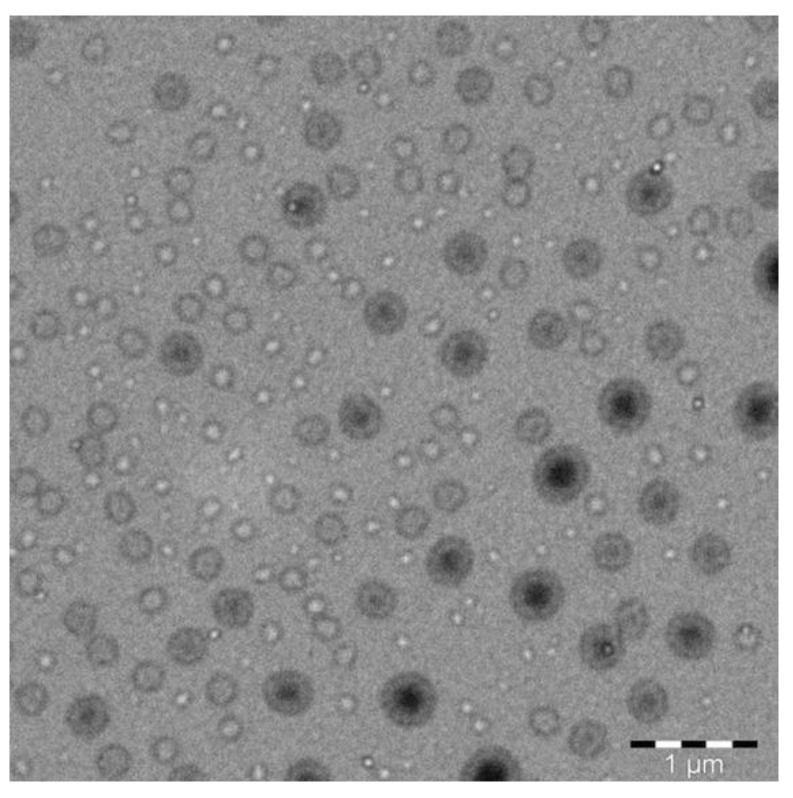
Transmission electron microscopy (TEM) for optimized SNEDDS F9 showing the droplets shape and size in submicron range.

The physicochemical characterization of APR-SNEDDS was carried out on the most stable formulations (F3, F4, F7, F8 and F9) by testing the following parameters: droplet size, polydispersity index (PDI), zeta potential (ZP), refractive index (RI), percentage of transmittance (%T) and surface morphology by transmission electron microscopy (TEM); but the later one was for the optimized APR-SNEDDS only. The results for the physicochemical characterization of APR-SNEDDS are included in Table 4. In terms of droplet size, all the results recorded were below 25 nm, which indicated high formulations uniformity and stability. In general, the droplet size was found to be reduced as the percentage of oil phase decreased in the formulation (15% oil phase in formulation F7, F8 and F9 that had the lowest droplet size). The mean droplet size was lowest in formulation F9 (17.505 ± 0.247 nm), which might be due to the presence of the highest percentage of S_mix_ ratio in the formulation (60%) that provided relatively high solubilizing capacity. The lowest PDI was 0.109 ± 0.019 in formulation F3 and the highest PDI was 0.278 ± 0.014 in formulation F8. The ZP values were negative for all formulations, F3 = −11.2, F4 = −17.4, F7 = −20.55, F8 = −17.65 and F9 = −13.35 mV, which was due to the composition of (o/w) APR-SNEEDS that presented the negatively charged molecules at the surface, due to the presence of the fatty acid esters in Lauraglycol-FCC (oil phase). The negative charges created repulsive forces between the nanoemulsion droplets, which reflected on the physical stability of the formulations in terms of the absence of droplets combination or phase separation that resulted in the clear and transparent appearances of the formulations [18]. The mean RI of the formulations was 1.340, which in the case of SNEDDS formulation meant that the formulation is of isotropic nature. The %T of the formulations was measured to determine their clarity/transparency translated into their ability to transmit the light rather than absorbing or blocking it. All the results recorded were ≥ 95%; formulation F3 had the lowest %T = 95.94% and formulation F9 had the highest %T = 99.15%. From the above findings, formulation F9 was selected as the optimum APR SNEDDS, based on its lowest droplet size (17.505 ± 0.247), relatively low value of PDI (0.147 ± 0.014) and ZP (−13.35 mV), average RI (1.337) and highest %T (99.15 ± 0.131). Therefore, the TEM analysis of its surface morphology was carried out and the results are presented below in Figure 2. The shape of the droplets was spherical and their size was ≤50 nm.

### 2.7. In Vitro Drug Release Studies

**Figure 3:**
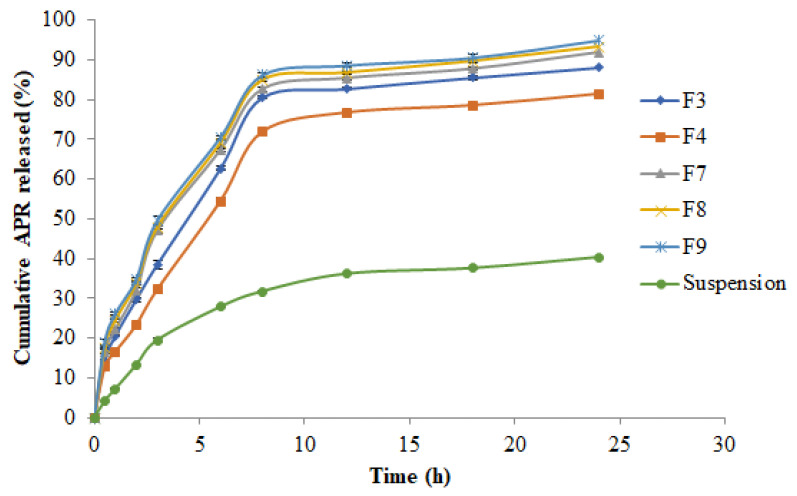
Cumulative in vitro release of APR from prepared APR-SNEDDS and APR suspension via dialysis membrane over 24 h.

The results of in vitro drug release studies are presented in Figure 3. The drug release pattern from APR-SNEDDS and APR-suspension was immediate and rapid but with APR-SNEDDS having a greater percentage of drug release during the first hours of the study compared to APR suspension. During the first 3 h of the study, APR-SNEDDS released more than 30% drug compared to the APR-suspension that released only 19.49%. Both formulations continued to release the drug gradually until the steady state was reached at 8 h. By that time, the cumulative drug release for formulations F3, F7, F8 and F9 was >80% but for formulation F4 it was 76.69% and for APR suspension, it was 31% only. As the study continued, the cumulative drug release from APR-SNEDDS and APR suspension continued to increase steadily until the end of the study (24 h). At 24 h, the cumulative drug release from F7, F8 and F9 exceeded 92% with F9 having the highest percentage of cumulative drug release (94.919% ≈ 95%). While, F4 had the least drug release compared to the other SNEDDS, reaching 81.36% cumulative drug release, followed by the APR suspension that had its maximum observed cumulative drug release throughout the entire study with a value of 40%. The ascending order for the cumulative drug release for the formulations at the 24 h time point was as follows: APR suspension = 40.3%, F4 = 81.36%, F3 = 88.11%, F7 = 91.80% and F8 = 93.20% and F9 = 95%. From these results, APR-SNEDDS F9 was selected for optimization and further investigation. 

### 2.8. Kinetic Analysis of Drug Release Data

Different kinetic models regarding the mechanism of drug release were studied, including zero-order model, first-order model, Higuchi model, the Hixon–Crowell model and the Korsemeyer–Peppas model [21,22,23]. The correlation coefficients (R^2^) and kinetic of drug release from APR-SNEDDS (F3, F4, F7, F8 and F9) and APR suspension are shown in Table 5. The different values of R^2^ indicated the best model fit the drug release pattern from each formulation. The F3 formulation had R^2^ = 0.999 following zero order release kinetics and Hixon–Crowell kinetics too. The best model fit for F4 was zero order kinetics with R^2^ = 0.999. For formulations F7, F8 and the optimum formulation F9, the highest R^2^ values were 0.997, 0.996 and 0.9995, respectively, fitting Hixon–Crowell drug release kinetics. For APR-suspension, the best model fitting its drug release kinetic was the Higuchi model with R^2^ = 0.996.

### 2.9. Bioavailability (In Vivo) Study and Pharmacokinetic Evaluation 

**Figure 4:**
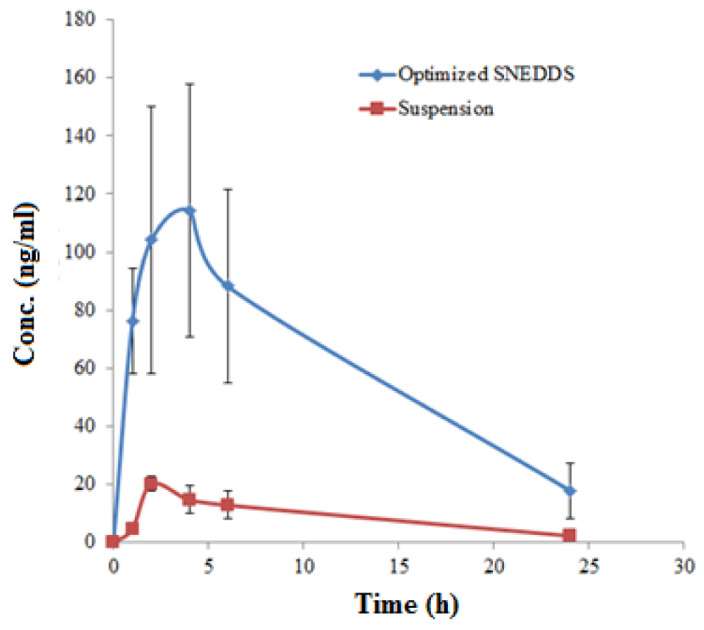
Plasma concentration–time plots of APR after oral administration of optimized SNEDDS and APR suspension in rats (Mean ± SD; *n* = 6; dose 3 mg/kg).

The analysis of APR in male rat plasma samples was performed using a UHPLC-MS/MS method as reported in the literature [24]. The concentration of APR in rat plasma samples was obtained using a calibration curve plotted between the concentration of APR and area ratio of APR to an internal standard (IR). The calibration curve of APR was found to be linear in the concentration range of 1.47–350 ng/mL. Both formulations showed immediate and rapid drug release during the first two hours of the study with the suspension reaching its maximum concentration of 20 ng/mL, but the F9 formulation continued to release drug sharply with a concentration of 103 ng/mL by that time. After two hours, the drug release from the suspension decreased gradually, reaching 0 ng/mL concentration at the end of the study (24 h). APR-optimized SNEDDS continued to increase readily after two hours until the maximum concentration of 119 ng/mL was reached at 5 h. After that, it decreased steeply, reaching 90 ng/mL concentration at 6 h. The next hours showed a gradual decrease in the drug plasma concentration until the end time point was reached (24 h) with a concentration of 22 ng/mL. Overall, the release profile of APR from optimized SNEDDS F9 was significant compared to the drug suspension (*p* < 0.05). The comparative in vivo APR release after oral administration of optimized SNEDDS and APR suspension are shown in Figure 4.

The results of each pharmacokinetic parameter (mean ± SD) of APR after an oral administration of optimized formulation (F9) and APR-suspension (3 mg/kg) are given in Table 6.

## 3. Discussion

Psoriatic arthritis is a progressive inflammatory disease that can lead to persistent inflammation, irreversible joint damage and disability. Current treatment options for psoriatic arthritis are limited because they lack optimal efficacy, and are mostly inconvenient for patients as they are quite expensive, involve injections, and are associated with serious adverse events. APR is the most recently approved oral anti-psoriatic arthritis drug and has been found superior to conventional treatment choices in adult patients with active disease. APR is classified as class IV according to the biopharmaceutics classification system (BCS). Class IV drugs are characterized by their low solubility and poor permeability, which affects their dissolution and absorption [8,9]. The lipid formulation approach appears as a promising approach that can be utilized for improving the solubility, dissolution properties and oral bioavailability of poorly soluble drugs [25,26,27]. SNEDDS were reported in many studies as the best formulation to solve the problems associated with class II, III and IV drugs, which have poor solubility or/and poor permeability, which affects their overall oral bioavailability [12,13,14,15,16,17,18]. This anhydrous formulation can rapidly form fine oil-in-water nanoemulsions upon dispersion in the gastrointestinal fluids under mild agitation imparted by the gastric motility [18]. Formation of submicron droplets upon dilution produce a large interfacial surface area for transfer of the drug, which may result in increased rate and extent of absorption and hence, improved bioavailability [12]. These formulations maintain the drug in a dissolved state throughout the GI tract and therefore, may enhance the bioavailability of poorly soluble drugs, for which absorption is dissolution rate limited [17,18]. To our knowledge, very limited approaches have been reported in the literature to enhance APR solubility, dissolution, permeability and oral bioavailability [10,11]. Therefore, the aim of this study was to enhance APR’s drawbacks using a less complicated and more reproducible method, which was perfectly achieved through the application of SNEDDS as a potential drug delivery system for APR. The current research was meant to enhance APR’s therapeutic efficacy by improving its in vitro rate of dissolution, solubility, and bioavailability. APR SNEEDDS were developed using a spontaneous emulsification method via the construction of pseudo-ternary phase diagrams, while different thermodynamic tests were carried out on the developed SNEDDS based on centrifugation, heating and cooling cycles, and freeze-pump thaw cycles. Subsequently, the thermodynamically stable SNEDDS were characterized by self-nanoemulsification efficiency, droplet size, PDI, ZP, RI, %T and surface morphology. The optimized SNEDDS of APR were then used for in vivo evaluation followed by statistical analysis.

The equilibrium solubility data of solutes in different components at room temperature or physiological body temperature was the technique that was applied for selecting the components to develop the suitable SNEDDS [18]. Screening of components by carrying out equilibrium solubility studies using the shake flask method [28] was the very first and the most important step in APR-SNEDDS fabrication, and determined the most suitable components for SNEDDS formulation. Their selection was also made upon their safety; they fall under GRAS category and their acceptability for oral pharmaceutical use. APR is a class IV drug; it is poorly soluble in water and its water solubility as a mole fraction at room temperature and atmospheric pressure is 2.74 × 10^−7^ as per EMA and USFDA [8,9]. The equilibrium solubility of APR in different SNEDDS components was found to vary significantly with the maximum equilibrium solubility being observed in Transcutol-HP, followed by Tween-80. These two components were used as surfactant and cosurfactant in APR-SNEDDS. The combination of a surfactant with a cosurfactant in the formation of o/w nanoemulsions with improved levels of solubilization greatly reduced interfacial tension and decreased interface fluidity [13]. The process of selection of the surfactant and cosurfactant in the further study was governed by the efficiency of emulsification and the solubilization ability of APR. In general terms, the surfactant was selected with emphasis being placed on the continuous phase of the nanoemulsion with the hydrophilic surfactant for nanoemulsion with the aqueous phase as the phase of dispersion and vice-versa [29,30]. The maximum oil solubility of APR was found in Lauraglycol-FCC and hence selected as the oil phase for formulation development. The oil solubility of the drug is crucial for its stability in the formulation and throughout the ingestion process in the GIT. It maintains the drug in the solubilized form, which prevents its precipitation that hinders its desirable solubility and absorption. The solubility studies were meant to identify the most preferred oil phase and surfactant to cosurfactant ratio for the development of the APR SNEDDS formulation. It was also observed in the literature that creating a distinction between the most suitable oil and surfactant to cosurfactant ratio that has the maximal solubilizing potential for the drug under investigation was essential, as it would lead to the improvement of the drug loading [18]. The results for solubility studies also showed that the least equilibrium solubility was observable with water, ethanol and IPA. The variation exhibited was significant and this can be explained based on the poor hydrophilicity of APR. This implies that APR is a molecule whose interactions with water and other polar substances are more favorable thermodynamically as compared to the interactions with either ethanol or IPA. The rule of thumb here is that the solubility of APR molecules in water is more than 1 mass percentage as long as the condition of having at least one neutral hydrophilic group for each 5 carbons is met or at least a single electrically charged hydrophilic group for each of the 7 carbons is met. As such, APR seems to attract water out of air. However, water was the most preferred solvent in the aqueous phase in APR SNEDDS considering that it is inert in nature, while it has other properties like high miscibility with formulation components and high formulation compatibility [19,20]. The self-emulsion formulations made up of oil, surfactant, cosurfactant and the drug should be clear and monophasic liquid at ambient temperature upon addition to the aqueous phase, while the solution should have good solvent properties [13]. The solubility studies were assessed in further pseudo-ternary phase diagrams interpretation and formulation development. Pseudo-ternary phase diagrams showed that lipophilic drugs like APR are preferably solubilized in the o/w nanoemulsions, while the w/o systems seem to be the better option for the hydrophilic drugs [29,30]. The loading of the drug at each formulation was found to be the most critical design factor in developing the nanoemulsion systems for the drug, considering that it is poorly soluble, which depends on the drug solubility at different formulation components. The formulation volume was minimized to the highest possible values to allow for the delivery of the therapeutic dose of the drug in the encapsulated form. In the case of oral formulation development, the drug solubility in the oil phase is of particular importance. The reasoning here is that the ability of the nanoemulsion to maintain APR in solubilized form was greatly dependent on the solubility of the drug in oil phase (Lauraglycol-FCC). Whenever the surfactant or cosurfactant was found to contribute to the drug solubilization, then it was easy to conclude that there is a high risk of precipitation since the dilution of the NEs in the gastrointestinal tract can contribute to the lowered solvent capacity of either the surfactant or cosurfactant [15]. Another suggestion is that it is essential to have a sound understanding of the factors that influence the capacity of drug loading while ensuring that the capability of the system is maintained to undergo the monophasic dilution with water, while also ensuring that the tendency for drug precipitation and crystallization is minimal [16]. Large amounts of surfactants were found to cause gastrointestinal and skin irritation upon oral and topical administration. This implies that the proper selection of the surfactants was essential, where it was essential to determine the surfactant concentration properly and use the minimum concentration in the formulation. Nonionic surfactants were also found to be less toxic as compared to the ionic counterparts, where they were also observed from the literature to have lower CMCs [31,32]. Furthermore, o/w nanoemulsion dosage forms for oral and parenteral use based on the nonionic surfactants were more likely to offer better in vivo stability [31]. The cosurfactant is a characteristic component in naoemulsions and an essential entity that is meant to maintain nanoemulsion systems at low surfactant concentrations [18]. The cosurfactant was observed to increase the mobility of the hydrocarbon tail, while it allowed greater penetration of the oil into the region. Alcohols (O-H) are reported to increase the miscibility of the aqueous and oil phases, bearing in mind that they tend to partition between the phases. Therefore, since the maximal solubility of APR was observed in Lauraglycol-FCC as compared to other oils, the nanoemulsion area was applied as the criteria for assessment and evaluation of the cosurfactants [18]. The pseudo-ternary phase diagrams were developed for APR-SNEDDS based on the spontaneous emulsification or aqueous phase titration method [31,32]. These phase diagrams were developed in order to optimize the APR SNEDDS. The size of the nanoemulsion regions in the phase diagrams was compared at an interchangeable S_mix_ ratio with the major measures including keeping the surfactant at the same levels while altering the cosurfactant and vice versa. There were six diagrams that were constructed, each consisting of three plots with each plot representing the different phases of the formulation. In the first plot, it is observed as the oil phase comprising of Lauraglycol-FCC, while the other plot was for the aqueous phase comprising of the water. The third phase was for the S_mix_ ratio comprising Tween-80: Transcutol-HP. In the course of formation of the S_mix,_ Tween-80 and Transcutol-HP were mixed together in several mass ratios from 1:0 to 4:1. On the other hand, the oil phase Lauraglycol-FCC was mixed in ratios of 1:9 to 9:1 with the S_mix_ ratios. After this, there was a drop-wise titration for the oil-S_mix_ ratios done by the water. The aim of creation of phase diagrams was to examine the maximum nano-emulsification. The research was based on the observation that the larger the size of the field of nano-emulsification, the greater the nano-emulsification efficiency of the system. It was also observed that whenever the length of chain was increased, there was an increase in the area of existence of the nanoemulsion. As such, the nanoemulsion formation was a function of the composition of the system, where the existence of the nanoemulsion formation was illustrated with the help of the pseudo-ternary phase diagram. In as much as the order of the mixing of the various components did little in terms of influencing the formation of the nanoemulsion, the system was kept at a thermodynamically stable condition that was path-independent. The other major observation was that there was no distinct conversion from the w/o to the o/w NEs. The rest of the region of the phase diagram was representative of the turbid and conventional emulsions. There was also careful observation of the formulations to ensure that the metastable systems were not selected, even though the free energy that was consumed in the formation of the NEs was very low, while the formation was thermodynamically spontaneous. For further optimization of the system, the effect of the surfactant and cosurfactant ratio on nanoemulsion formation was determined. A total of six phase diagrams were developed with each containing different ratios of the aqueous phase, oil phase, and the S_mix_. Based on the S_mix_ ratios, the first phase diagram with the (1:0) S_mix_ ratio portrayed the least nano-emulsification areas, while the phase diagram with the (1:2) S_mix_ ratio portrayed very small nano-emulsification areas too. For the phase diagrams with (1:1) and (2:1) S_mix_ ratios, the maximal nano-emulsification areas were observed with the diagram with the S_mix_ ratio of (1:1) portraying a superior phase diagram. The other phase diagrams with (3:1) and (4:1) S_mix_ ratios showed moderate nano-emulsification areas as compared to the least emulsification areas (1:0) and (1:2). From the results obtained, the phase diagram with the (1:1) S_mix_ ratio and the largest nano-emulsification areas was selected for the APR-SNEDDS formulation development. It was concluded that whenever the cosurfactant is absent or present at lower concentrations, the surfactant cannot have the potential of sufficiently reducing the o/w interfacial tension. An o/w NEs region was found towards the rich apex of the phase diagram. The maximum concentration of oil that could be solubilized as shown in the phase diagram was at 66% of S_mix_. Whenever the cosurfactant was added to the surfactant within equivalent amounts, a higher nanoemulsion region was exhibited. The increase in the nanoemulsion region relative to the addition of the surfactant is attributed to the reduction in the interfacial tension and increased fluidity of the interface at S_mix_ [18]. The selection of the phase diagram with the (1:1) S_mix_ ratio for the APR-SNEDDS formulation development was based on the observation that the higher the nanoemulsion field is, the greater the nanomulsification efficiency of the system. In the current research, the (1:1) S_mix_ ratio of Tween 80 and Transcutol-HP gave the maximal nano-emulsification area, which was selected for the development of the nine APR-SNEDDS (F1-F9). Each of the mixtures contained 5 mg of APR in a total volume of 1 mL. The preparation of the formulations was based on the entire range of SNEDDS zones in the phase diagram with varied levels of the oil phase concentrations with Lauraglycol-FCC being given a preference and the deionized water or aqueous phase concentrations being applied. APR as the drug under investigation was allowed to dissolve completely in Lauraglycol-FCC before the S_mix_ (Tween 80 and Transcutol-HP) was added to the mixture, followed by titration with deionized water. For the purpose of exclusion of the possibility of metastable formulations, thermodynamic stability tests were carried out. Most of the representative formulations were extracted from the o/w nanoemulsion region of the phase diagram, which was constructed at an S_mix_ ratio of (1:1) as it was observed to show the largest nano-emulsification areas for the APR-SNEDDS formulations development. Thermodynamic stability tests were carried out on the nine formulated APR-SNEDDS for the exclusion of the metastable and unstable formulations by the application of various external conditions that could impact on the stability. Formulations including F3, F4, F7, F8, and F9 were found to withstand the tests, as they portrayed no lack or loss of stability in terms of phase separation and drug precipitation. Thermodynamic stability was a measure that could aid in conferring the long shelf life to the nanoemulsion as compared to the ordinary emulsion [16]. The formulations were prepared based on the nature of the entire range of the SNEDDS zones in the phase diagram with various Lauraglycol-FCC (oil phase) concentrations of 10, 15, 20, 25% *v*/*v*, the S_mix_ concentrations of 30, 40, 45, 50, 55, 65% *v*/*v*, and aqueous phase concentrations of 25, 30, 35, 40, 50, 55 *v*/*v*. The most stable formulations (F3, F4, F7, F8, and F9) were subjected to the physicochemical characterization with tests aiming at understanding the effect of the droplet size, PDI, ZP, RI, %T and surface morphology. In the first test of the droplet size, all the results were recorded to be below 25 nm, which was an indicator that there was high formulation uniformity and stability. The droplet size was heavily dependent on the oil phase formulation, where the droplet size was reduced related to the decrease in the percentage of the oil phase formulation with 15% oil phase formulation F7, F8, and F9 having the lowest droplet size readings. The mean droplet size was approximately 23.517 nm with the lowest result being recorded in formulation F9 that recorded 17.505 nm. The reduced droplet size can be explained in terms of the presence of the highest percentage of S_mix_ ratio in the formulation (60%), which provided relatively high solubilizing capacity. Studies have shown that the droplet size distribution is one of the most essential characteristics that affect the in vivo fate of NEs as it influences the bioactive release rate and absorption. The production of NEs with smaller droplet sizes is highly recommended as it provides extremely low surface tension for the entire system and the interfacial tension of the o/w droplets [17,18]. The mean PDI for APR-SNEDDS was reported as 0.182, which is an indication of the narrow size distribution and more uniformity of the droplets within the formulations. Smaller particles tend to resist gravity separation, flocculation, coalescence, and creaming. The ZP values were also found to be negative for all formulations F3 (−11.2 mV), F4 (−17.4 mV), F7 (−20.55 mV), F8 (−17.65 mV), while F9 was −13.35 mV. The charge difference among different formulations was possible due to different compositions of different formulations. The significant charge differences between the formulations F3 and F7 could be possible due to the high concentration of S_mix_ in formulation F7 compared to formulation F3. The explanation behind the observation is attributed to the composition of the o/w APR-SNEDDS that presented negatively charged molecules at the surface due to the presence of fatty acid esters in the Lauraglycol-FCC (oil phase). The negative charges were also found to create repulsive forces between the nanoemulsion droplets, which reflected the physical stability of the formulations in terms of the absence of droplet combinations or phase separation that resulted in the clear and transparent appearances of the formulations. The %T of the formulations was also considered as an essential component that would aid in the determination of their clarity/transparency translated into their ability to transmit the light as opposed to absorbing or blocking it. All the results recorded were less than or equal to 95% with formulation F3 having the lowest %T (95.94%) and formulation F9 having the highest %T (99.15%). From the physicochemical characterization of APR-SNEDDS, formulation F9 was selected as the optimum APR SNEDDS, considering that it has the lowest droplet size, and relatively low value of PDI and ZP, while it had the highest %T. 

The in vitro drug release studies were carried out to investigate the release profile of APR from the APR SNEDDS that were stable including F3, F4, F7, F8, and F9 and the APR-suspension over a period of 24 h though a dialysis membrane. The results show that within the first three hours of the study, APR-SNEDDS released more than 30% of the drug as compared to the APR-suspension that released only 19.49%. From the cumulative in vitro release of APR from prepared APR-SNEDDS and APR suspension over a period of 24 h, it can be said that there are variations because of the changes in the suspension agent that affected the drug release pattern from the suspension formulation compared to the superior APR-SNEDDS (F9) formulation. The results show that the formulation and process parameters in the preparation of NE containing APR is critical in obtaining the desirable attitudes for effective drug delivery. Different kinetic models regarding the mechanism of drug release were studied, including the zero-order model, first-order, Higuchi model, Hixon–Crowell model and Korsemeyer–Peppas model [21,22,23]. The different values of R^2^ indicated the best model fit the drug release pattern from each formulation. The F3 formulation had R^2^ = 0.999 following zero order release kinetics and Hixon–Crowell kinetics too. The best model fit for F4 was zero order kinetics with R^2^ = 0.999. For formulations F7, F8 and the optimum formulation F9, the highest R^2^ values were = 0.997, 0.996 and 0.9995, respectively, fitting Hixon–Crowell drug release kinetics. For APR-suspension, the best model fit its drug release kinetic was the Higuchi model with R^2^ = 0.996. The different pattern of the drug release model in different formulations could be possible due to the presence of different concentrations of oil phase and S_mix_. For the in vivo drug release, comparisons were made on rat plasma concentrations of the optimized APR-SNEDDS (F9) compared to that of the APR suspension. In male rats, the plasma concentration of APR after oral administration is too low [11]. The HPLC method is not able to detect the low concentration of APR in plasma. The UPLC-MS/MS method is a very sensitive method, which is able to detect the low concentration of APR in rat plasma. Hence, the UPLC-MS/MS method was used to determine APR in rat plasma [24]. Both formulations were found to portray immediate and rapid drug release during the first two hours of the study with the suspension reaching its maximum concentration at 20 ng/mL, while the F9 formulation continued to release drug sharply with a concentration of 103 ng/mL by that time. After a period of two hours, the drug release from the suspension decreased gradually, reaching 0 ng/mL concentration at the end of the study (24 h). While APR-optimized SNEDDS continued to increase readily after two hours until the maximum concentration of 119 ng/mL was reached at 5 h. After that, it decreased steeply, reaching 90 ng/mL concentration at 6 h. The next hours showed a gradual decrease in the drug plasma concentration until the end time point was reached (24 h) with a concentration of 22 ng/mL. Overall, the release profile of APR from optimized SNEDDS F9 was significant compared to the drug suspension (*p* < 0.05). The noncompartmental pharmacokinetic model was used to calculate different pharmacokinetic parameters of APR including C_max_, AUC_0–t_, AUC_0–inf_, λz, T_½,_ T_max_ and relative bioavailability [33,34,35]. The most significant parameters compared to the APR suspension were the T_max_ = 4.00 ± 0.96 h, which was 2.0 ± 1.70 h for the APR suspension, AUC_0–t_ = 3256.76 ± 212.50 ng.h/mL compared to APR suspension 462.83 ± 52.25 ng.h/mL, AUC_0–∞_ = 3481.04 ± 235.51 ng.h/mL compared to APR suspension 488.13 ± 61.31 ng.h/mL and the relative bioavailability = 703.66% compared to APR suspension = 100%, which indicated a seven-fold increase in APR bioavailability (*p* ˂ 0.05). 

## 4. Materials and Methods

### 4.1. Materials

APR was purchased from Beijing Mesochem Technology Pvt. Ltd. (Beijing, China). From Gattefossé (Lyon, France), Lauroglycol-90, Capryol-90, Labrasol, Capryol-PGMC, Transcutol-HP, Labrafil-M1944CS, Lauroglycol-FCC, Labrafac-PG and Peceol were purchased. Ethanol, isopropyl alcohol (IPA), polyethylene glycol-400 (PEG-400), ethylene glycol (EG), propylene glycol (PG), Tween-80, Triton-X100 and Tween-85 were acquired from Sigma Aldrich (St. Louis, MO, USA). Cremophor-EL was acquired from BASF (Cheshire, UK). From Nikko Chemicals (Tokyo, Japan), Sefsol-218 and HCO-60 were obtained. HPLC-grade solvents, Ethanol Chromasolv^®^ absolute for HPLC was purchased from Sigma Aldrich (St. Louis, MO, USA) and methanol HPLC-grade was purchased from Fischer Scientific (Waltham, MA, USA). Lastly, from ELGA water purification system (Wycombe, UK), the deionized water was procured.

### 4.2. Screening of Components

The equilibrium solubility of APR was examined in different oils (Triacetin, Lauroglycol-90, Lauroglycol-FCC, Capryol-90 and Capryol-PGMC), surfactants (Tween-80, Labrasol, Cremophor-EL and Triton-X100), cosurfactants (Transcutol-HP, PEG-400, ethanol, PG, EG, IPA and water). The water is frequently used as an aqueous phase, as found in the literature [19,20]. The method used to confirm the saturated solubility of APR was the equilibrium method [28]. The solubility of APR in each component was determined at 25 °C. The excess amount of solid APR was added in known amounts of each component in triplicates. Each mixture was vortexed for about 5 min and transferred to the “OLS 200 Grant Scientific Biological Shaker (Grant Scientific, Cambridge, UK)” at the shaking speed of 100 rpm for the period of 72 h [9]. After 72 h, each mixture was removed from the biological shaker, and filtered and centrifuged at 5000 rpm. The supernatants were taken, diluted suitably with mobile phase (wherever applicable) and subjected for the analysis of APR content using RP-HPLC method at 254 nm. The concentration of APR in solubility samples was determined by a calibration curve of APR.

### 4.3. Construction of Pseudo-Ternary Phase Diagrams for APR SNEDDS

Nanoemulsions are multicomponent systems and therefore, pseudo-ternary phase diagrams are most suitably constructed for them. After choosing the SNEDDS components from the solubility studies, the pseudo ternary phase diagrams are constructed. Generally, phase diagrams are graphical plots that are used to examine different thermodynamic parameters of a given system. They show the relationship between the different system phases at equilibrium or even various conditions. They identify the factors that could affect the equilibrium such as temperature, pressure, concentration and pH. The number of plots is related to the number of the components in a system. In the case of SNEDDS, phase diagrams identify the emulsification areas of the nanoemulsion and the number the plots on the phase diagram is three. One plot is for the oil phase, the second plot is for the aqueous phase, and the third one represents the surfactants mixture ratio (S_mix_ ratio). In APR SNEDDS, the aqueous phase used was de-ionized water, the oil phase was Lauroglycol-90, the surfactant was Tween-80, and the cosurfactant was Transcutol-HP. To form the S_mix_, Tween-80 and Transcutol-HP were mixed together in several mass ratios from 1:4 and 4:1 ratios. Then, the oil phase Lauroglycol-90 was mixed from 1:9 to 9:1 ratios with the S_mix_ ratios. After that, gradual or drop wise titration for the oil-S_mix_ ratios by the de-ionized water was carried out, refereeing this step as phase titration. The appearance of each mixture was observed in terms of clarity and turbidity during the titration. A clear transparent appearance stood for nanoemulsion and a turbid milky appearance stood for regular emulsion. Finally, the physical observations were marked on the phase diagram to note the optimum ratios for the further APR SNDDS formulation [30,31,32].

### 4.4. Formulation Development

Using the aqueous phase titration method/spontaneous emulsification method to create the phase diagrams, the maximum SNEDDS zones for APR-SNEDDS were identified [31,32]. The maximum SNEDDS zones were observed with 1:1 mass ratio of Tween-80 and Transutol-HP. Nine APR-SNEDDS in a total of 1 mL each were utilized using the 1:1 S_mix_ ratio, namely F1, F2, F3, F4, F5, F6, F7, F8 and F9, considering almost an entire range of SNEDDS zones in the phase diagram with various Lauroglycol-FCC concentrations (10, 15, 20, 25% *v*/*v*), S_mix_ concentrations (30, 40, 45, 50, 55, 60% *v*/*v*), and aqueous phase concentrations (25, 30, 35, 40, 50, 55% *v*/*v*). Each formulation contained 5 mg of the drug dissolved completely in Lauroglycol-FCC before the addition of Tween-80 and Transcutol-HP mixture followed by vortex shaking until a clear and transparent mixture was obtained. The deionized water was added gradually by a drop wise pattern while vortexing with different concentrations used for each formulation to produce particulate free and clear formulations. The composition of each formulation is illustrated in Table 2.

### 4.5. Thermodynamic Stability Testing

The purpose of thermodynamic stability testing is to exclude metastable APR-SNEDDS (stable under certain conditions and/or reform slowly) and unstable APR-SNEDDS (unstable under standard conditions and/or do not reform) through applying various external conditions that might affect their stability, such as centrifugation, heating–cooling cycles and freeze–pump–thaw cycles. The centrifugation of APR-SNEDDS (F1–F9) was carried out at 5000 rpm, 25 °C for 30 min, the heating–cooling cycles were carried out between 4 °C (refrigerator) and 50 °C (oven) for 48 h for 3 cycles, and the freeze–pump–thaw cycles were carried out between −21 (freeze) and +25 °C (thaw) for 24 h for 3 cycles [36,37].

### 4.6. Self-Nanoemulsification Efficiency Test

The APR-SNEDDS that withstood the thermodynamic stability testing were subsequently subjected to self-nanoemulsification efficiency testing. The aim of this test is to examine the SNEDDS stability regarding the occurrence of phase separation or precipitation upon dilution with water. In order to conduct this test, the dilution of 1 mL from each APR SNEDDS was done 500 times with different diluents, such as 0.1 N HCl, deionized water and phosphate buffer (pH 6.8). The efficiency of each SNEDDS was evaluated and graded via inspection and the use of a system for grading [12,16]:

Grade A: Rapidly forming clear/transparent nanoemulsion (emulsify within 1 min) 

Grade B: Rapidly forming bluish white nanoemulsion (emulsify within 2 min) 

Grade C: Milky emulsions (take more than 2 min to emulsify)

Grade D: Dull, grayish milky emulsions (take more than 3 min to emulsify) 

Grade E: Emulsions with oil globules at the surface (take more than 5 min to emulsify).

### 4.7. Physicochemical Characterization

Developed APR SNEDDs were physicochemically characterized by testing a number of variables such as droplet size, PDI, ZP, RI, %T, and surface morphology [37,38,39]. For the droplet size measurement, 1 drop of APR-SNEDDS was diluted with water at 25 °C with a scattering angle of 90° using Malvern Particle Size Analyzer (Malvern Instruments Ltd., Holtsville, NY, USA). PDI was measured using the same dilution, temperature, scattering angle and instrument as the droplet size measurement. Additionally, ZP of diluted APR-SNEDDS with water was measured using glass electrodes at pH 7.0. The RI was measured by Abbes Refractometer without any sample dilution. The %T was estimated for 1 drop APR-SNEDDS diluted with methanol and a blank of methanol using a spectrophotometer at 550 nm detection wavelength. Lastly, the surface morphology evaluation of optimized APR-SNEDDS was performed using the dilution by TEM at 100–200 Kv.

### 4.8. In Vitro Dissolution Studies

The purpose of this study was to develop a comparison between in vitro APR release from the developed APR-SNEDDS (F1-F9) and APR suspension. The investigation was carried out using a dialysis membrane from Spectrum Medical Industries (Mumbai, India; MWCO 12,000 Da) and dissolution apparatus in accordance with United States Pharmacopoeia (USP) XXIV method [18] with the following conditions: rotational speed fixed at 100 rpm in 500 mL dissolution media of pH controlled 6.8 phosphate buffer, at 37 ± 0.5 °C temperature. An amount of 1 mL from both the APR SNEDDS and APR suspension was transferred to the dialysis bags. From each formulation, a 3 mL sample was withdrawn at regular time intervals and replaced at the same time with 3 mL of drug free dissolution media (phosphate buffer pH 6.8). The amount of APR in each sample was determined using the reported RP-HPLC method [8]. The drug release mechanism from the SNEDDS formulation was studied via the application of different kinetic models such as zero order, first order, Higuchi, Hixson–Crowell, and Korsemeyer–Peppas models [21,22,23].

### 4.9. Bioavailability Study and Pharmacokinetic Evaluation

A single oral dose parallel-built study was performed for a bioavailability and pharmacokinetic study on twelve male Wistar Albino rats weighing around 200–250 kg, provided from the Animal Care and Use Centre, College of Pharmacy, King Saud University, Riyadh, Saudi Arabia. The entire study was performed in accordance with King Saud University Animal Care and Use Committee guidelines and were approved by the Animal Ethical Committee of King Saud University (Approval number: SE-19-123). Before starting the experiment, the rats were acclimatized in plastic cages under common lab conditions, maintaining controlled temperature and humidity of 25 ± 2 °C and 55 ± 5% RH, respectively, with a light/dark cycle (12 h), drinking water, and feeding on rats’ pellet diet ad libitum. The rats were randomly divided into two groups (*n* = 6 in each group), which served as APR suspension (sodium carboxymethyl cellulose, 0.5% *w*/*v*) and optimized APR SNEDDS (F9) treatment groups, respectively. The rats were fasted overnight before the experiments. Blood samples (approximately 500 µL) were taken from the retro-orbital plexus into heparinized microfuge tubes at 0, 1, 2, 4, 6 and 24 h after oral administration of APR (3 mg/kg, oral) in both groups. Plasma samples were harvested by centrifuging the blood at 5000× *g* for 8 min. Plasma samples were mixed with acetate buffer (pH 4.6) in the ratio of 1:10 (buffer: plasma) and stored in a deep freezer at −80 ± 10 °C until further analysis.

The drug analysis was carried out using a reported UPLC-MS/MS method [24]. A validated and reported UPLC-MS/MS (UPLC, Waters Acquity, Milford, MA, USA) was employed to determine the concentration of APR in rat plasma [24]. The chromatographic conditions involved the use of a Acquity BEH C18 column (100 mm × 2.1 mm, 1.7 μm), mobile phase mixture (85:15, *v*/*v*) of acetonitrile and 10 mM ammonium acetate and flow rate of 0.30 mL/min. The eluted compounds (APR and IS) were detected by tandem mass spectrometry using TQ detector (Waters Corp., Milford, MA, USA) attached to an electrospray ionization (ESI) source operating in negative ionization mode. A protein precipitation method with the use of ethyl acetate as a solvent were carried out for the extraction of the drug from rat plasma. In this study, celecoxib was used as the IS. About 20 μL of IS combined with 200 μL of rat plasma (2.0 μg/mL) and 2.0 mL of ethyl acetate. The mixture was vortexed for 2.0 min. The samples were centrifuged at 50,000 rpm for about 5 min and 500 μL of the supernatant was removed and placed in a sample vial for further analysis in the UPLC-MS/MS system. The analysis of APR in rat plasma was carried out via the injection of about 5 μL sample into UPLC-MS/MS.

The plasma concentration values of APR at different time intervals were used to evaluate its pharmacokinetic profiles by plotting drug concentration–time curves. The software used for calculation of pharmacokinetic parameters of APR was WinNonlin software (Pharsight Co., Mountain View, CA, USA) [35]. The noncompartmental pharmacokinetic model was used to calculate the C_max,_ T_max_, AUC_0–t_, AUC_0–inf_, λz and T_½_ [40,41].

### 4.10. Statistical Analysis

Diverse physicochemical variables, drug delivery and biological data was analyzed using GraphPad InStat^®^ software (San Diego, CA, USA) applying unpaired Dunnett’s test. Differences between each two related parameters were considered statistically significant for a *p*-value of ≤0.05.

## 5. Conclusions

APR is the first orally administered drug approved for the treatment of active psoriatic arthritis, which is a painful and inconvenient disease that can lead to other diseases and disability. In comparison to the common treatment regimens, the treatment with APR was well tolerated by the patients and associated with better overall disease-related improvements. In terms of side effects, it was found that APR has minimal adverse events but only upon the initiation of therapy, and they can be resolved or controlled during the treatment course. However, the marketed film-coated immediate release tablets of APR Otezla^®^ have 20–33% bioavailability compared to an APR absolute bioavailability of 73%. To our knowledge, very limited approaches to enhance APR solubility, dissolution, permeability and oral bioavailability have been reported in the literature. Therefore, the aim of this study was to enhance APR’s drawbacks using a less complicated and more reproducible method. This was perfectly achieved through the application of SNEDDS as a potential drug delivery system for APR. SNEDDS were reported in many studies working as solvers for the problems associated with class II, III and IV drugs, which have poor solubility or/and poor permeability, which affects their overall oral bioavailability. In conclusion, the optimum formulation was F9, composed of 15% oil, 60% S_mix_, and 25% aqueous phase with the lowest droplet size (17.505 ± 0.247 nm), low PDI (0.147 ± 0.014), low ZP (−13.35 mV), highest %T (99.15 ± 0.131), maximum in vitro drug release (94.9%) over 24 h and optimum relative bioavailability (703.66%). Following the promising results of the current study, future studies should be carried out to evaluate the major factors that influence the encapsulation efficiency and stability of APR-containing NEs and the application of the formulations for the oral delivery of APR.

## Figures and Tables

**Table 1:** Equilibrium solubility values of apremilast (APR) in different oils, surfactants, cosurfactants, and water at 25 °C (mean ± SD, *n* = 3).

Components	Solubility ± SD (mg/mL)
Water	0.01 ± 0.00
Ethanol	0.66 ± 0.01
IPA	2.07 ± 0.10
EG	7.21 ± 0.52
PG	7.96 ± 0.64
Triacetin	11.42 ± 0.95
PEG-400	12.36 ± 0.28
Capryol-90	18.15 ± 1.03
Capryol-PGMC	21.41 ± 1.32
Lauroglycol-90	28.21 ± 1.45
Cremophor-EL	33.81 ± 2.04
Lauraglycol-FCC	36.54 ± 2.78
Labrasol	37.54 ± 2.14
Triton-X100	41.24 ± 3.12
Tween-80	48.54 ± 3.76
Transcutol-HP	55.01 ± 3.19

**Table 2:** Composition of 1 mL APR-SNEDDS each containing 5 mg of the drug.

Code	Oil (%)	S_mix_ (%)	Water (%)	Total (mL)
F1	10	40	50	1 mL
F2	15	40	50	1 mL
F3	20	40	40	1 mL
F4	25	40	35	1 mL
F5	15	30	55	1 mL
F6	15	45	40	1 mL
F7	15	50	35	1 mL
F8	15	55	30	1 mL
F9	15	60	250	1 mL

**Table 3:** Results for self-nanoemulsication and thermodynamic tests.

SNEEDS	Self-Nanoemulsication Test Grade	Thermodynamic Tests		
		CENT.	H&C	FPT
F1	A	√	√	M
F2	A	√	√	M
F3	A	√	√	S
F4	A	√	√	S
F5	A	√	√	Un.
F6	A	√	√	M
F7	A	√	√	S
F8	A	√	√	S
F9	A	√	√	S

CENT.: centrifugation, H&C: heating-cooling cycle, FPT: freeze-pump thaw cycle, M: metastable, S: stable, Un.: unstable, √: passed the test.

**Table 4:** Physicochemical characterization of APR-SNEDDS.

SNEDDS	Characterization Parameter ± SD				
	Droplet Size (nm)	PDI	ZP (mV)	RI	%T
F3	24.95 ± 0.169	0.109 ± 0.019	−11.2	1.343 ± 0.001	95.94 ± 0.221
F4	37.07 ± 2.234	0.237 ± 0.070	−17.4	1.341 ± 0.000	96.6 ± 0.222
F7	18.725 ± 0.275	0.139 ± 0.022	−20.55	1.341 ± 0.001	96.67 ± 0.128
F8	19.335 ± 0.021	0.278 ± 0.014	−17.65	1.339 ± 0.001	97.25 ± 0.022
F9	17.505 ± 0.247	0.147 ± 0.014	−13.35	1.337 ± 0.001	99.15 ± 0.131

SD: standard deviation, PDI: polydispersity index, ZP: zeta potential, mV: millivolts, RI: refractive index; %T: percentage of transmittance.

**Table 5:** The correlation coefficients and kinetics of APR release from SNEDDS and suspension.

Formulation	Zero Order		First Order		Higuchi	Hixon-Crowell	Korsemeyer-Peppas	
	$K_{0\;}$	R^2^	$K_{1\;}$	R^2^	R^2^	R^2^	R^2^	n
F3	0.115	0.999	10.037	0.964	0.975	0.999	0.996	1.600
F4	0.127	0.999	9.936	0.978	0.983	0.994	0.987	1.547
F7	0.113	0.992	10.353	0.951	0.995	0.997	0.995	1.671
F8	0.112	0.994	10.783	0.954	0.995	0.998	0.996	1.737
F9	0.112	0.993	11.217	0.955	0.995	0.997	0.995	1.803
Suspension	0.262	0.975	7.747	0.897	0.996	0.987	0.993	1.324

**Table 6:** Pharmacokinetic parameters of APR after an oral administration of optimized SNEDDS and APR suspension (3 mg/kg) in rats.

Parameters	APR Suspension (Mean ± SD)	SNEDDS (Mean ± SD)
C_max_ (ng/mL)	20.19 ± 2.59	114.17 ± 43.42
T_max_ (h)	2.0 ± 1.70	4.00 ± 0.96 *
AUC_0–t_ (ng.h/mL)	462.83 ± 52.25	3256.76 ± 212.50 *
AUC_0–∞_ (ng.h/mL)	488.13 ± 61.31	3481.04 ± 235.51 *
λz (h^−1^)	0.09 ± 0.01	0.08 ± 0.01
T_½_ (h)	7.70 ± 1.28	8.66 ± 2.18
Relative bioavailability (%)	100	703.66 *

* *p* ˂ 0.05 significant compared to APR suspension.

## Data Availability

This study did not report any data.

## Sample Availability

Samples of the compounds APR are available from the authors.
